# Microbial Biosurfactants: A Bridge from Aquatic Environments to Subsurface Oil Recovery: Mechanisms, Challenges, Prospects

**DOI:** 10.3390/life16030484

**Published:** 2026-03-16

**Authors:** Jing Chang, Wei Yang, Yulin Jin, Zhichao Zhou, Zhaoxi Song, Wei Zhao, Shizhen Liang, Yanfang Ma

**Affiliations:** 1 College of Chemistry and Chemical Engineering, Xi’an University of Science and Technology, Xi’an 710054, China; jchang@xust.edu.cn (J.C.); 23415030213@stu.xust.edu.cn (W.Y.); zhaowei3859520@163.com (W.Z.); 22415030123@stu.xust.edu.cn (S.L.); 2 Dalian Petrochemical Company, China National Petroleum Corporation, Dalian 116031, China; jinyulin@petrochina.com.cn (Y.J.); zhouzc_dl@petrochina.com.cn (Z.Z.); 3 Dongxin Oil Production Plant of Shengli Oilfield, Dongying 257094, China; songzx416.slyt@sinopec.com; 4 Key Laboratory of Comprehensive and Highly Efficient Utilization of Salt Lake Resources, Qinghai Institute of Salt Lakes, Chinese Academy of Sciences, Xining 810008, China

**Keywords:** microbial biosurfactants, wettability alteration, microbial-enhanced oil recovery (MEOR), aquatic extremophiles

## Abstract

Microbial biosurfactants, derived from diverse aquatic and extreme ecosystems, offer a sustainable and environmentally compatible strategy for enhanced oil recovery by fundamentally altering subsurface rock wettability. These biologically produced amphiphiles can efficiently transform oil-wet rock surfaces into water-wet states, thereby mobilizing otherwise trapped crude oil. The primary aim of this review is to provide an integrative understanding of how these biomolecules function at the interface between aquatic microbial ecology and subsurface petroleum engineering, with a particular focus on wettability alteration as a key mechanism for enhancing oil recovery. This review surveys major biosurfactant classes—glycolipids, lipopeptides, and polymeric bioemulsifiers—and their core mechanisms, emphasizing their relevance to challenging reservoir conditions such as high temperature and salinity. A detailed assessment is devoted to persistent hurdles such as stability, adsorption onto rock formations, and economic scalability. Future prospects center on three key approaches: advancing synergistic “bio-hybrid” systems that integrate biosurfactants with complementary agents such as biopolymers and nanomaterials; achieving cost-effective production through the valorization of waste feedstocks; and expanding targeted bioprospecting of microbial diversity from extreme aquatic environments. Together, these strategies are reviewed to drive the advancement of robust, green microbial-enhanced oil recovery (MEOR) technologies, charting a course from fundamental insights to field-scale implementation.

## 1. Introduction

For the Earth’s systems, water resources serve as the primary integrator, connecting the atmosphere, lithosphere, and biosphere through the hydrological cycle. In these aquatic environments, microbial communities function as micro-scale engines that drive biogeochemical reactions across the entire planet [1]. The vast variability of these habitats—from oligotrophic groundwater and high-salinity subterranean brines to glaciers and thermal springs—fosters an immense diversity of microbial life. Microorganisms in these settings are not just passive inhabitants, but can interact with and ultimately alter their surroundings. Notably, extremophiles such as thermophilic and halophilic bacteria, often isolated from deep oil reservoirs, saline aquifers, or geothermal environments, exhibit remarkable resilience to the harsh conditions similar to subsurface petroleum systems [2,3]. Through their metabolic activities, these microbes drive essential biogeochemical cycles. Harnessing this microbial multifunctionality is paramount for developing novel biotechnologies. This review explores how microorganisms from diverse aquatic environments provide a biotechnological solution for enhancing oil recovery while protecting subsurface water resources, bridging fundamental microbial functions to sustainable application [4].

Relentless growth in global energy demand—driven by population growth, industrialization, and digitalization including AI infrastructure—continues to pressure petroleum resources. Following a 5.8% surge in global energy consumption in 2021 [5], electricity demand is forecast to grow over 3% annually through 2026 [6]. As this demand persists, enhancing recovery from existing oil reservoirs becomes critically significant. Currently, the average global recovery rate is only 30–40% [7,8], and a substantial portion (often 50–70%) of the original oil in place (OOIP) is retained within the reservoir [9]. This is because most of the world’s oilfields are mature, having undergone primary recovery (driven by natural reservoir pressure) and secondary recovery (typically waterflooding) methods. Unlocking this vast, untapped resource is a strategic priority. The low recovery stems from the fact that primary recovery extracts only 5–10% of the OOIP, while subsequent secondary recovery can yield an additional 10–40%, leaving a significant fraction unrecovered [10,11,12,13,14,15,16]. This residual oil is held by strong capillary forces and is often adhered to the rock surfaces in reservoirs that have become “oil-wet” over millions of years of contact with crude oil. Consequently, simply injecting more water becomes highly inefficient, as the water channels through the more permeable, water-wet paths, bypassing the trapped oil. This necessitates the use of Enhanced Oil Recovery (EOR) techniques to mobilize and produce this remaining resource.

For decades, Chemical Enhanced Oil Recovery (cEOR) has been a primary strategy to mobilize residual oil trapped in mature reservoirs [17,18]. cEOR encompasses all methods that modify the chemical composition of injected water, either by adding agents like polymers, surfactants, alkalis, nanofluids, ionic liquids, or mutually soluble solvents (e.g., Dimethyl Ether), or by engineering the water’s ionic composition itself [19]. These chemicals function by reducing interfacial tension, altering wettability, or improving sweep efficiency, typically increasing recovery factors to between 30% and 40% [7,8]. Despite various field-proven cEOR methods being implemented worldwide [20,21,22,23,24,25,26,27], their widespread deployment is hindered by significant economic, operational, and environmental challenges [28]. Subsurface performance is often compromised under harsh reservoir conditions, as synthetic polymers and surfactants can degrade, adsorb onto rock, and block pore throats [18,29,30,31]. Production and post-production stages present further difficulties, including stable emulsions, scaling and corrosion, and the complex disposal of toxic chemicals [32,33,34]. More importantly, many synthetic surfactants are toxic and recalcitrant, posing a long-term threat of groundwater contamination towards subterranean aquatic ecosystems [35,36]. These multifaceted limitations underscore the urgent demand for alternative EOR technologies that are more sustainable, cost-effective, and operationally robust.

**Figure 1:**
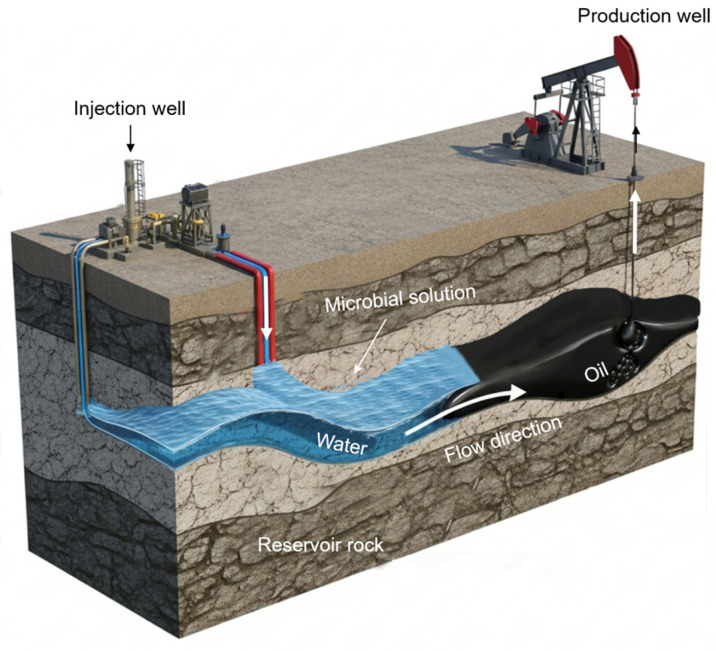
Schematic of microbial-enhanced oil recovery (MEOR) integrated with subsequent waterflooding to displace oil [4].

Under these circumstances, Microbial-Enhanced Oil Recovery (MEOR), as illustrated in Figure 1, emerges as an ideal eco-friendly alternative to address these challenges. By harnessing natural processes, it aligns with the global drive for sustainable technology [37]. MEOR takes advantages of the metabolic activities of microorganisms, which can be injected into the reservoir (in situ) or cultivated externally (ex situ) to produce valuable metabolites that can improve residual oil recovery [38]. Bacteria, in particular, are favored in MEOR due to their durability under extreme subsurface conditions and their ability to produce valuable metabolic products such as biopolymers, bio-gases, and crucially, biosurfactants [39,40]. The potential of MEOR technology is substantiated by numerous field trials conducted globally, including extensive applications in the United States [38,41]. Among these metabolites, microbial biosurfactants are of paramount interest. These naturally derived, amphiphilic molecules offer profound advantages over synthetic chemical surfactants: they are highly biodegradable, low-toxicity, groundwater-friendly, and are often produced by extremophilic microorganisms that can adapted to high-temperature and high-salinity (HTHS) conditions similar to oil reservoirs [3]. A critical mechanism for biosurfactants in MEOR is wettability alteration: by adsorbing at the rock–oil interface, they dislodge crude oil and shift the surface from oil-wet to water-wet, mobilizing trapped residual oil by lowering capillary forces [42,43,44]. Thus, microbial biosurfactants uniquely bridge biological processes from aquatic environments to subsurface engineering, positioning them as pivotal agents for sustainable recovery strategies.

This review is motivated by the need to bridge the gap between the promising laboratory performance of microbial biosurfactants and their limited field-scale implementation. To map out their journey from discovery to application, this review seeks to demystify their central role in oil recovery by focusing on the pivotal wettability alteration mechanism—an understanding essential to explaining their effectiveness in harsh reservoir environments. While clarifying that this core mechanism illuminates the path forward, this review also critically assesses the substantial practical barriers—from microbial resilience in subsurface conditions to economic viability—that currently stand between scientific promise and widespread implementation. By weaving together the current frontier of knowledge, this review aim to provide a forward-looking framework that bridges fundamental science and engineering practice, with the hope of inspiring researchers to transform these natural, adaptive molecules into cornerstone technologies for a more sustainable energy transition—solutions that draw upon biological ingenuity to mitigate environmental impact.

## 2. Mechanisms of Biosurfactant-Based Microbial-Enhanced Oil Recovery in Reservoirs

Biosurfactants are amphiphilic molecules produced by microbial secondary metabolism. This structure, featuring both hydrophilic and hydrophobic moieties, provides them with the capability of lowering interfacial tension between immiscible phases. This intrinsic property accounts for their widespread utility across diverse industrial and environmental fields. In petroleum industry, the potent surface-active property of biosurfactants are harnessed for applications ranging from bioremediation and oil spill dispersion to storage tank cleaning and heavy metal desorption [45], with microbial-enhanced oil recovery (MEOR) representing a particularly critical application. The secret of MEOR’s feasibility lies in the ability of microbial metabolites to modify subsurface physicochemical conditions by acting at rock–oil–water interfaces, thereby mobilizing otherwise trapped oil [3].

Based on their chemical moieties, biosurfactants are broadly categorized into groups including lipopeptides, glycolipids, phospholipids, fatty acids, polymeric surfactants, and particulate surfactants [46,47,48]. The functional properties of biosurfactants vary with their molecular size: low-molecular-weight (MW) biosurfactants excel at reducing interfacial tension, while high-MW biosurfactants are particularly effective at forming stable emulsions [45,49,50].

These fundamental colloidal and interfacial properties allow biosurfactants to mobilize trapped crude oil in MEOR through primary mechanisms including interfacial tension reduction, wettability alteration, and emulsification. The mobilization of trapped oil and the ultimate residual oil saturation are fundamentally controlled by the capillary number ($N_{c}$), a key dimensionless group in displacement efficiency that represents the ratio of viscous to capillary forces [4,51,52], as defined in Equation (1):

(1) $$N_{c}=\frac{\mathrm{viscous}\;\mathrm{force}}{\mathrm{capillary}\;\mathrm{force}}=\frac{v\mu }{\sigma \cos\theta }$$

where v is the velocity of the displacing fluid (in ms), μ is its viscosity (in Pa·s), σ is the interfacial tension (IFT) between the displaced (oil) and displacing (e.g., water) fluids (in N/m), and θ is the contact angle (in degrees) quantifying the wettability of the rock surface. A higher capillary number correlates with a lower residual oil saturation. Consequently, a primary objective of MEOR is to increase the capillary number by altering one or more parameters in Equation (1). Microbial biosurfactants contribute precisely to this end, primarily by directly lowering the interfacial tension (σ) and profoundly changing the rock wettability (thereby affecting cosθ), which synergistically enhances crude oil mobilization. The following sections will deconstruct these targeted mechanisms in detail, elucidating the interfacial science that positions microbial biosurfactants as a uniquely powerful tool for sustainable hydrocarbon recovery.

### 2.1. Interfacial Tension Reduction

A fundamental property of any surfactant, including those of microbial origin, is the ability to reduce the tension at the interface between two immiscible liquids, in this case, oil and water. In a reservoir, residual oil is trapped in pore throats by capillary forces, which are directly proportional to the oil–water IFT. High IFT creates a strong energy barrier that prevents the oil from being deformed and squeezed through the narrow pore channels by the displacing fluid (water) [42].

**Figure 2:**
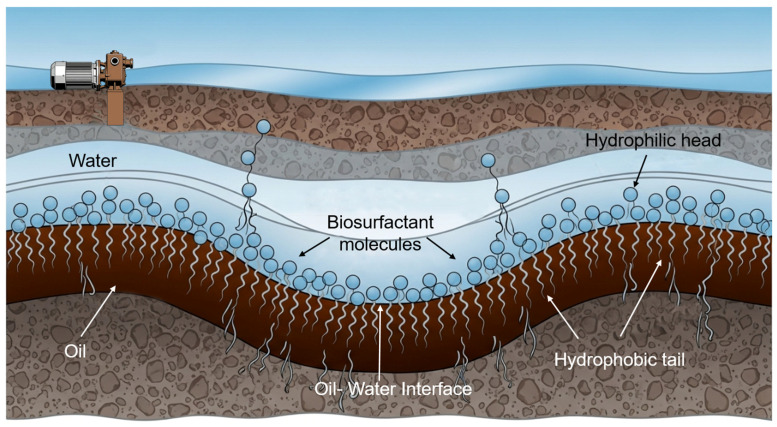
Schematic of biosurfactant accumulation and orientation at the oil–water interface informed by representations in [42].

Biosurfactants, being amphiphilic, naturally accumulate at the oil–water interface. Their hydrophobic tails penetrate the oil phase, while their hydrophilic heads remain in the aqueous phase (see Figure 2). This arrangement disrupts the cohesive forces between water molecules at the interface, leading to a dramatic reduction in IFT. By lowering the IFT, biosurfactants reduce the capillary pressure required to mobilize an oil droplet, allowing it to be displaced by the hydrodynamic force of the injected water [53].

Numerous studies have verified the efficacy of this IFT reduction mechanism in both laboratory and core-flooding contexts. For instance, Bacillus subtilis SL produces a surfactin-type biosurfactant that reduces the oil–water IFT to about 20 mN/m when using it alone. After mixing with the chemical anionic surfactant, the mixed system lowered the IFT against crude oil to 0.95 ± 0.22 mN/m under optimal salinity conditions. Furthermore, the fermentation solution of this strain obtained an additional 5.66% in crude oil recovery during core flooding tests under low-permeability reservoir conditions [54].

The lipopeptide biosurfactant produced by Bacillus licheniformis L20, when mixed with a chemical anionic surfactant (1,3-diacylglycerols-2-sulfate), can reduce the crude oil–water IFT to 0.109 mN/m at an optimal NaCl concentration. Core flooding experiments showed that this anionic surfactant/biosurfactant mixed system could increase the oil recovery rate by 24.12% after waterflooding [55].

A complex formed by mixing microbial surfactin (LS-Bl) with the chemical surfactant polyethylene glycol tert-octyl phenyl ether (Tn) at a 7:3 ratio significantly reduces the IFT between brine and heavy crude oil to 0.2 mN/m at 60 °C. Flooding tests using this complex surfactant solution at a concentration of 6 g/L achieved a total oil recovery of 59.21% in a sand-packed model, demonstrating its effectiveness in mobilizing residual oil [42].

Early research in MEOR primarily attributed the efficacy of biosurfactants to IFT reduction, analogous to the mechanism of chemical surfactants. Nevertheless, a consensus in the published literature indicates that biosurfactants generally fail to obtain the ultra-low IFT (typically below 10^−2^ mN/m) required for effective chemical flooding. While most microbial products can moderately lower IFT, they seldom reach the ultra-low levels necessary for significant incremental oil recovery [56]. Notably, recent laboratory studies have demonstrated that under specific conditions, certain microbial processes can indeed achieve this critical ultra-low IFT threshold (see Table 1), revealing new potential for biosurfactant applications.

This leads to a critical qualification regarding the role of IFT reduction. While IFT reduction is a crucial first step, in strongly oil-wet reservoirs, its effect is limited if the oil remains strongly adhered to the rock surface. Therefore, IFT reduction is most effective when it acts in concert with the more dominant mechanism of wettability alteration [44].

### 2.2. Wettability Alteration: The Primary Driver of Recovery

Wettability describes the preference of a solid surface to be in contact with one fluid rather than another. For an oil reservoir, it is the affinity of the rock surface (e.g., sandstone or carbonate) for either oil or water [67,68,69,70]. It is widely documented and acknowledged that a thin layer of water coats the primary reservoir rock surfaces. Consequently, most reservoir rocks initially demonstrate inherent water-wet characteristics [71]. In mature reservoirs, the inherent water-wet nature of rock surfaces can be changed to oil-wet or mixed-wet states over time [72]. The adsorption of polar components (e.g., asphaltenes and resins) from crude oil onto mineral surfaces is the primary driver of this wettability reversal, which overrides the initial water-wet state. Among these, asphaltenes exhibit a more pronounced effect on inducing an oil-wet state in sandstones compared to resins. The adsorption of these components shows a strong preference for clay minerals (e.g., chlorite) over quartz, as clays provide favorable sites for interaction with polar organic compounds, leading to the formation of stable clay–organic complexes that render the surface oil-wet [73].

**Figure 3:**
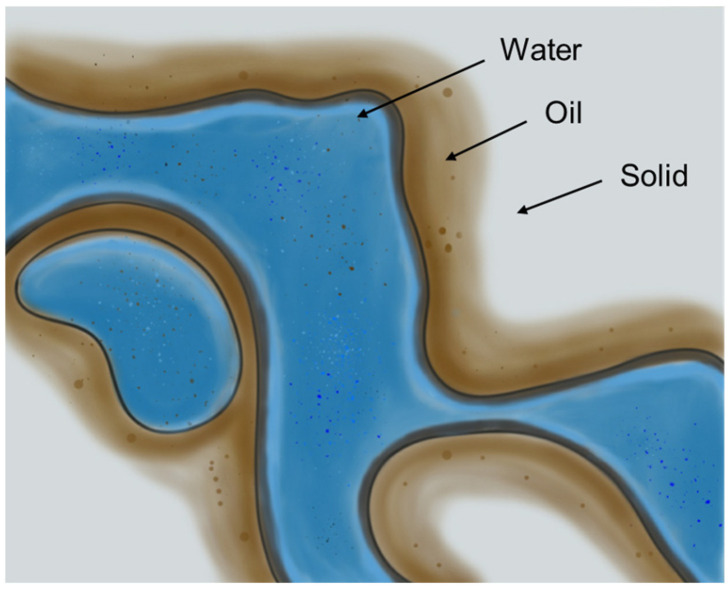
Fluid distribution in initial oil-wet condition in an untreated micromodel (brown: oil, blue: water phase, and white: solid); reproduced from [74].

In oil reservoirs with such mixed-wet situation, oil prefers to form a continuous film over the oil-wet surfaces (especially in larger pores), while water is constrained in pore corners or regions that retain water-wet properties (see Figure 3) [74]. During secondary waterflooding, the injected water preferentially flows through the more permeable, interconnected water-wet network of pores and throats, bypassing the large volumes of oil that adhered to the oil-wet rock surfaces. This preferential flow, a direct consequence of wettability alteration, results in poor sweep efficiency, early water breakthrough, and high residual oil saturation, ultimately limiting the overall recovery factor.

The paramount function of biosurfactants in these reservoirs is to reverse this condition, i.e., to alter the rock surface from oil-wet to water-wet [3], which can fundamentally change the fluid distribution and flow dynamics, improving oil recovery. Among the three major mechanisms mentioned above, wettability alteration is especially pivotal in mature oil reservoirs; some analyses suggest that in certain applications, wettability alteration alone may account for over 50% of the incremental oil recovery—more than the sum of all other mechanisms [44].

The function of biosurfactants in altering wettability is not uniform, which is affected by specific reservoir conditions, primarily salinity and the hydrophile–lipophile balance (HLB) of the biosurfactants. This is supported by detailed studies: in distilled water, high-HLB surfactin achieves a strongly water-wet state, while rhamnolipid with low-HLB can only induce a neutral-wet state; conversely, in seawater, rhamnolipid becomes effective in creating a strongly water-wet condition, whereas surfactin loses its ability. This conditional efficacy highlights the necessity of tailoring biosurfactant selection to the reservoir’s ionic environment for optimal results [43].

Given biosurfactants’ central role, complex mechanism and condition-dependent nature, a thorough explanation of how biosurfactants reverse wettability is therefore essential.

#### 2.2.1. Mechanisms of Wettability Reversal

**Figure 4:**
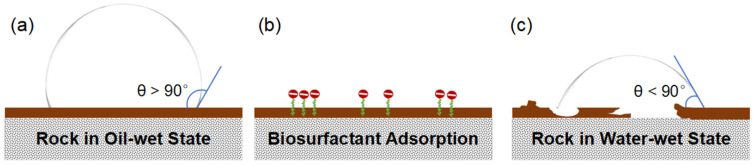
Mechanism of wettability reversal by biosurfactants from oil-wet to water-wet. (**a**) Initial oil-wet state (*θ* > 90°); (**b**) biosurfactant adsorption at the interface; (**c**) oil film displacement and final water-wet state (*θ* < 90°) [75].

The alteration in wettability by biosurfactants is primarily an adsorption-driven process at the three-phase (rock–oil–water) contact line. The general mechanism can be visualized as follows (Figure 4):

- Initial State: In an oil-wet system, polar organic components from crude oil firmly adhere onto the rock surface, creating a hydrophobic layer. Oil spreads across this surface, the water phase contact angle (*θ*) is greater than 90°.
- Biosurfactant Introduction: When a biosurfactant solution is introduced, the amphiphilic molecules migrate to the interfaces.
- Adsorption and Displacement: The biosurfactant molecules then adsorb onto the rock surface. The specific interaction depends on the nature of the biosurfactant and rock mineralogy. The hydrophobic tail of the biosurfactant can interact with the adsorbed oil layer via hydrophobic interactions, while the hydrophilic head has an affinity for the water phase. In many cases, the biosurfactant can insert itself between the rock surface and the adsorbed oil film, effectively “lifting off” or displacing the organic material.
- Formation of a Water-wet Layer: As biosurfactants accumulate at the surface, they orient themselves with their hydrophilic head groups facing outwards into the aqueous phase. This creates a new, stable hydrophilic layer on the rock surface.
- Final State: The rock now preferentially contacts water. The oil film retracts into discrete droplets, and the contact angle drops below 90°. Consequently, water can spontaneously penetrate finer pore networks, thereby mobilizing the trapped oil and improving the overall sweep efficiency of the waterflood. A study showed that wettability alteration alone could be responsible for an oil production increase of over 50% in a mature well, boosting overall recovery from around 32% of OOIP to over 62% in MEOR cases [44].

#### 2.2.2. Adsorption Behaviors on Different Rock Surfaces

The wettability alteration efficiency greatly differs with the interactions between the biosurfactant and the specific mineralogy of the reservoir rock. The two most common reservoir rock types, sandstones and carbonates, present different surface chemistries.

- Carbonate Surfaces (Limestone, Dolomite): Carbonate rocks are typically composed of calcite (CaCO_3_) and dolomite (CaMg(CO_3_)_2_). In reservoir brines, their surfaces often carry a positive charge. Anionic biosurfactants (which are common), such as the glycolipid rhamnolipid and the lipopeptide surfactin, possess negatively charged carboxylate groups (-COO^−^) in their hydrophilic heads. This leads to strong electrostatic attraction between the biosurfactant and the positively charged carbonate surface, promoting robust adsorption and effective wettability alteration [43]. Nevertheless, this interaction can be complex. High salinity, particularly the presence of divalent cations (Ca^2+^, Mg^2+^) in seawater or formation brine, can screen the electrostatic charges or lead to biosurfactant precipitation, potentially inhibiting its effectiveness.
- Sandstone Surfaces (Quartz, Feldspars, Clays): Sandstone reservoirs are primarily composed of quartz (SiO_2_), with varying amounts of feldspars and clays. Under typical reservoir pH conditions, these silicate minerals carry a negative surface charge. Consequently, anionic biosurfactants would experience electrostatic repulsion, hindering their adsorption. In such cases, adsorption is driven by other forces like hydrophobic interactions between the biosurfactant tail and any adsorbed organic matter, or van der Waals forces. A major challenge in sandstone reservoir is the significant biosurfactant loss through adsorption onto clays, which possess high surface area and complex charge distribution. Non-ionic or cationic biosurfactants may be more effective in these formations, although they are less commonly studied for MEOR. Despite this challenge, biosurfactants demonstrably alter sandstone wettability: contact angle measurements on sandstone slices treated with a microbial consortium show a decrease from >120° (oil-wet) to 60° (water-wet) within 3–4 days, corresponding to a huge capillary pressure change of ~5000 Pa, sufficient to increase oil recovery via spontaneous imbibition [44]. This mechanistic gap was later addressed by Zihui Chen et al. [75], whose AFM and molecular simulation study elucidated the distinct actions of two key biosurfactants, providing a quantitative and structural explanation for their differing efficacies. They reported that rhamnolipid reduces the adhesion force between oil and glass by adsorbing onto the oil film and presenting hydrophilic head groups outward, thereby lowering surface hydrophobicity to create a weakly water-wet state. In contrast, surfactin can peel the oil film away from the rock surface and reverse the interaction force between oil and a glass surface from attractive to repulsive, leaving a strongly water-wet rock surface. The more effective and efficient wettability alteration capability of surfactin stems from its much higher adsorption energy (−38.01 eV) compared to rhamnolipid (−12.23 eV) on hydrophobic surfaces. This difference in the adsorption energy is attributed to their different molecular structures: the longer alkyl chain (C_12_) of surfactin strengthens hydrophobic–hydrophobic interactions, whereas shorter tails (C_7_) and the presence of a carboxyl group of rhamnolipid diminishes its hydrophobicity and adsorption stability. This fundamental insight confirms that overcoming sandstone’s mineralogical constraints requires biosurfactants with molecular structures specifically tailored for strong hydrophobic adsorption—directly linking nano-scale interactions to macroscopic recovery efficacy, which is critical for designing effective MEOR strategies.

### 2.3. Emulsification and Mobility Control

Beyond altering static surface properties, biosurfactants also influence fluid dynamics within the reservoir. A key mechanism is the in situ formation of oil-in-water (O/W) emulsions, which can improve oil recovery through mobility control.

**Figure 5:**
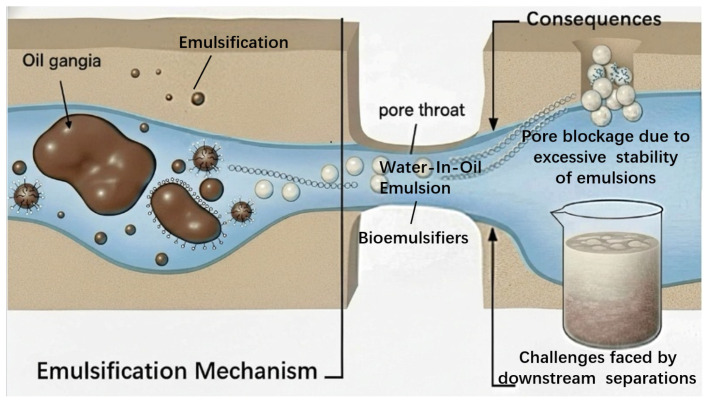
Schematic of oil-in-water emulsion formation in an oil reservoir [4].

- Emulsification: By drastically lowering IFT, biosurfactants can promote the formation of emulsions, typically O/W emulsions. These emulsions break down large, continuous oil ganglia into smaller, mobile droplets that can be more easily transported through the porous medium. High-MW polymeric biosurfactants, often called bioemulsifiers, are particularly effective at creating stable emulsions that can be flushed out of the reservoir [55]. Nevertheless, the formation of overly stable emulsions can be detrimental, causing pore plugging and posing challenges for downstream oil–water separation facilities (see Figure 5).
- Mobility Control: A successful waterflood requires favorable mobility control, meaning the displacing fluid (water) should not advance much faster than the displaced fluid (oil). An unfavorable viscosity ratio leads to “viscous fingering”, where water bypasses large regions of oil, severely reducing sweep efficiency [76]. During in situ MEOR applications, biosurfactants are usually not the only product generated by microorganisms; biopolymers like xanthan gum and scleroglucan can also be produced. The co-produced biopolymers improve the overall rheological behavior of the injected fluid via enhancing the viscosity of the injected water [77,78]. This enhances the mobility ratio and leads to a more stable displacement front and a much higher volumetric sweep efficiency, ensuring that the mobilized oil is effectively pushed towards the production wells. The synergy between biosurfactants mobilizing the oil and biopolymers improving the sweep is a powerful combination for maximizing recovery [4,79].

### 2.4. Complementary Roles of Other Microbial Products in Microbial-Enhanced Oil Recovery

While biosurfactants are central to wettability alteration and interfacial tension reduction, a successful MEOR process often relies on a suite of metabolic products that work synergistically. Understanding the multifaceted roles of these other microbial by-products is crucial for a holistic view of the technology.

- Organic Acids: Many microorganisms, such as Clostridium species, ferment carbohydrates to produce low-MW organic acids [4]. These acids play a dual role. First, they can dissolve carbonate minerals in the reservoir rock, a process often referred to as “acidizing,” which enlarges pore throats and increases rock permeability [4,10]. Second, the reaction of these acids with carbonates can generate significant volumes of CO_2_ in situ, contributing to reservoir repressurization and oil swelling [4,10].
- Biomass (Microbial Cells): The accumulation of microbial cells, or biomass, within the porous matrix can be strategically beneficial. By selectively plugging high-permeability “thief zones,” biomass can divert subsequent injection water into lower-permeability and unswept oil-rich layers, thereby improving macroscopic sweep efficiency [4,10]. Furthermore, microbial cells themselves can act as surface-active agents, adhering to oil droplets and contributing to oil emulsification. Their attachment to rock surfaces can also alter the local wettability, aiding in oil release [4].
- Gases (CO_2_, CH_4_, H_2_): Microbial metabolism produces various gases, with CO_2_ being the most significant for EOR [80]. The in situ generation of gases serves multiple functions: (1) Reservoir Repressurization: Gas accumulation increases reservoir pressure, providing a driving force to push oil towards production wells. (2) Oil Swelling and Viscosity Reduction: CO_2_ is highly soluble in crude oil, causing it to swell, significantly reducing its viscosity, which makes it more mobile [4,10]. (3) Increased Permeability: CO_2_ can dissolve in formation water to form carbonic acid, which, similar to organic acids, can react with and dissolve carbonate rocks, further enhancing permeability [4].
- Biosurfactants: As detailed in previous sections, biosurfactants are key to reducing oil–rock interfacial tension and facilitating emulsification. This action helps to mobilize trapped oil by forming stable oil-in-water emulsions and reducing the adhesive forces that bind oil to the rock surface.
- Biopolymers: Produced by microorganisms like Xanthomonas campestris and Bacillus species, biopolymers such as xanthan gum primarily function in mobility control [4,81]. By increasing the viscosity of the injected water, they improve the mobility ratio, suppress viscous fingering, and lead to a more stable displacement front [82]. In some cases, biopolymers can also contribute to selective pore plugging, similar to biomass, by forming biofilms that block high-permeability zones [10].

By integrating these complementary mechanisms, a robust MEOR strategy leverages the full metabolic potential of the microbial community, achieving a synergistic effect that surpasses the sum of its individual parts.

## 3. Biosurfactant Categories and Formulations

The vast microbial diversity discovered in aquatic and terrestrial environments provides a rich portfolio of biosurfactants with distinct structures and properties (see Table 2). Research has focused on identifying and optimizing specific classes of biosurfactants that are particularly well-suited for the demanding conditions of oil reservoirs. Molecular weight serves as a primary criterion for classifying biosurfactants: low-MW biosurfactants and high-MW bioemulsifiers [83,84]. Producers of low-MW biosurfactants (e.g., glycolipids and lipopeptides) include Bacillus, Pseudomonas, Rhodococcus, and Nocardia species [85,86,87]; whereas high-MW bioemulsifier (e.g., glycoproteins and lipopolysaccharides) are predominantly produced by Acinetobacter, Bacillus, and Geobacillus species [88,89]. These metabolic products—among which glycolipids and lipopeptides are the most prominent and extensively studied for MEOR owing to their superior surface activity, stability in harsh reservoir conditions, and higher production feasibility—play indispensable roles via multiple EOR mechanisms [88,90].

### 3.1. Glycolipids

Glycolipids are hydrophilic carbohydrates linked to hydrophobic fatty acid chains by ester bonds. They are among the most studied biosurfactants for environmental and industrial applications.

#### 3.1.1. Rhamnolipids

Primarily produced by Pseudomonas species, rhamnolipids are anionic glycolipids that have become a benchmark for biosurfactant performance. They are powerful surface-active agents capable of reducing the IFT between oil and water to very low values. Their most significant attribute for MEOR is their exceptional wettability alteration capability, effectively converting oil-wet calcite and silica surfaces to strongly water-wet conditions [43,75,91,94,95]. Furthermore, rhamnolipids exhibit outstanding stability across a broad spectrum of temperatures (40–121 °C), pH values, and salinities (up to 10% *w*/*v*), making them robust candidates for a wide range of reservoir conditions [91].

#### 3.1.2. Sophorolipids

Sophorolipids are produced mainly by yeasts like Starmerella bombicola, which exist in two different structure forms: an acidic (open-chain) form, which is a better detergent; and a lactonic (cyclic) form, which is more effective at reducing surface tension [96]. Although the wettability alteration ability of sophorolipids was less studied compared to rhamnolipids, their excellent emulsification properties and low-cost production potential make them promising agents for EOR [97].

### 3.2. Lipopeptides

Lipopeptides, produced predominantly by Bacillus species, are a class of powerful biosurfactants composed of a hydrophobic fatty acid chain linked to a hydrophilic peptide chain [98,99]. Cyclic lipopeptides (e.g., surfactin) are a prominent subclass of lipopeptides, which have potent bioactivity and stability due to their macrocyclic structure via the linkage of a lactone bond. Lipopeptides also have linear variants, and an amide bond typically forms the acyl linkage. This unique amphiphilic structure of lipopeptides allows them to excel at emulsification and interfacial tension reduction. Consequently, lipopeptides have drawn broad interest from various fields, from bioremediation to pharmaceuticals [66]. As for MEOR, their exceptional surface activity and tolerance to reservoir conditions make cyclic lipopeptides (notably surfactin and lichenysin) outstanding agents [100].

#### 3.2.1. Surfactin

Produced by various strains of Bacillus subtilis, surfactin is renowned for its exceptional surface activity. Its cyclic peptide structure confers high thermal stability [100]. Surfactin has demonstrated effectiveness in reducing IFT. For example, it has been reported to lower the surface tension of water from 72 mN/m to 27 mN/m at very low concentrations [3,101]. Surfactin is also good at altering the wettability of carbonate surfaces [43,101]. Nevertheless, its performance can be hindered in high-salinity brine environment, particularly with high concentration of calcium and magnesium, due to precipitation and adverse electrostatic interactions [43].

#### 3.2.2. Lichenysin

Lichenysin, a lipopeptide biosurfactant produced by Bacillus licheniformis, shares structural similarities with surfactin but demonstrates enhanced robustness under extreme conditions [55]. It exhibits exceptional stability across a wide range of salinities, temperatures, and pH values. Notably, studies have demonstrated that certain lichenysin variants were capable of reaching an ultra-low IFT of 0.006 mN/m against decane and a critical micelle concentration as low as 10 mg/L, which is among the lowest recorded for any surfactant [102]. This superior IFT reduction property makes lichenysin a highly promising agent for EOR in challenging reservoirs, such as offshore and hypersaline environments.

### 3.3. High Molecular Weight Bioemulsifiers

High-MW biosurfactants (also known as bioemulsifiers) are complex mixtures of polysaccharides, proteins, lipopolysaccharides, and lipoproteins, whereby proteins are usually the main emulsifying component [103,104]. The examples include Emulsan, Alasan, and Liposan [45]. In MEOR, their primary function is not IFT reduction like low-MW biosurfactants, but the formation of highly stable O/W emulsions, which are crucial for mobilizing viscous or heavy crude oils. They function by adsorbing tightly at the oil–water interface, forming a robust protective film that can prevent droplet coalescence. The formed low-viscosity, flowable emulsions facilitate the transport of viscous oils through a porous reservoir rock. A field trial demonstrated that bioemulsifier injection led to an incremental oil production of approximately 88 tons from a single well, with a dramatic decrease in water cut from 85% to 25% [105]. Furthermore, high-MW biosurfactants can also adsorb onto the aged reservoir rock surface and alter its wettability. In the oil industry, bioemulsifiers such as Emulsan can be used not only in subsurface recovery, but also in cleaning oil storage tanks and stabilizing crude oil emulsions for pipeline transportation [45].

### 3.4. Formulation of Biosurfactants

Although biosurfactantsan is an ideal eco-friendly choice for EOR, a major constraint in applying biosurfactants is the limited production capacity of native microbial strains. Scientists are turning to metabolic engineering and synthetic biology to overcome this hurdle. By precisely editing the gene clusters that govern biosurfactant synthesis (e.g., the srf operon for surfactin, rhl operon for rhamnolipids), it is more likely to develop engineered strains with superior performance [105]. The engineering strategies focus on several interconnected objectives.

The primary focus is increasing the yield of biosurfactants, which is mainly achieved by overexpressing key regulatory or biosynthetic genes to enhance both the rate and the final titer of production. Beyond improving yield, it is also essential to lower the cost of substrates by utilizing cheaper, non-food feedstocks like lignocellulose or industrial waste.

Simultaneously, lots of efforts have been made to improve the robustness of the producer strains, thus enhancing the tolerance to extreme subsurface oil reservoir conditions, including high temperature, high salinity, and the existence of toxic inhibitors. In addition, a growing number of research is focusing on customizing structures to create novel variants with tailored properties (e.g., different fatty acid chain lengths or peptide sequences) for specific reservoir conditions.

## 4. Synergistic Biopolymer and Biocomplex Formulations

While microbial biosurfactants offer a uniquely sustainable pathway for altering subsurface wettability and mobilizing residual oil, their standalone application in EOR can be constrained by factors such as poor macroscopic sweep efficiency and suboptimal interfacial activity under harsh reservoir conditions. To compensate for the limitations of microbial surfactants in field application, composite systems formulated with complementary agents are commonly employed. These composite systems are designed to create a multi-mechanistic displacement process where the biosurfactant’s core functions are amplified and integrated with mobility control and stability enhancement.

### 4.1. Synergy with Biopolymers: Integrating Pore-Scale Mobilization with Macroscopic Sweep

As mentioned earlier, a major challenge in any flooding process is poor sweep efficiency due to unfavorable mobility ratios [82]. While biosurfactants are excellent at mobilizing residual oil at the pore scale, their effect is diminished if the displacing fluid bypasses large sections of the reservoir. The co-injection of a viscosity-enhancing biopolymer addresses this macroscopic challenge [106].

- Role of Biopolymers: This macroscopic challenge is addressed by co-injecting biosurfactants with viscosity-enhancing biopolymers, such as xanthan gum or scleroglucan. These are high-MW polysaccharides produced by microorganisms—xanthan gum by the bacterium Xanthomonas campestris and scleroglucan by the fungus Sclerotium rolfsii., respectively. By significantly thickening the aqueous phase at low concentrations, they function as highly effective viscosifiers, improve the oil–water mobility ratio and establish a stable displacement front [107,108]. Their excellent shear-thinning behavior ensures injectability, while their relative stability at reservoir temperatures underpins their widespread use in EOR for reliable mobility control [109,110,111].
- Synergistic Mechanism: The composite system of biosurfactants and biopolymers creates a synergistic “release-and-sweep” mechanism. In the combined flooding process, biosurfactants work at the leading edge by reducing IFT and altering wettablility to remove the aged oil from rock surfaces, then biopolymers enhance the mobility of the displaced oil toward production well by increasing the viscosity of the injected fluid [112]. This synergy enhances both pore-scale displacement and reservoir-scale sweep efficiency, yielding significantly EOR results than using either agent alone [111,112,113].

### 4.2. Synergy with Nanoparticles and Chemicals: Augmenting Interfacial Activity and Stability

To further boost the interfacial performance of biosurfactants and address specific reservoir challenges, integration with nanomaterials or low-dose chemicals represents a cutting-edge frontier [114].

- Biosurfactant–Nanoparticle Hybrids: Recently, the combination of biosurfactants with nanoparticles (e.g., silica, ZnO) has become a research focus, as nanoparticles can synergistically improve the interfacial performance at both fluid–rock and fluid–fluid interfaces to a higher level [115,116]. Studies have shown that nanoparticles and biosurfactants could co-adsorb, and form a denser and more resilient film, thus achieving ultra-low IFT [117,118]. Azarshin et al. revealed that nanoparticles can generate structural disjoining pressure to peel oil films from rock surfaces, which accounts for their highly effective wettability alteration ability [119]. In the meantime, biosurfactants act as dispersants, preventing nanoparticles from aggregation and ensuring their propagation in deep reservoir. Furthermore, Yulong Liu et al. synthesized a promising biological nanocomposite fluid for offshore oilfield application with nano-Fe_3_O_4_, nano-Ag and biopolymer sodium alginate, and revealed that nanoparticles can interact with polymer chains in this tertiary formulations, enhancing thermal and mechanical stability of the entire displacing system [120]. The outstanding synergy effect was also confirmed by a recent work by Amr Gazem et al., who integrated ZnO nanoparticles with a rhamnolipid/sophorolipid blend and xanthan gum, which achieved 95.14% oil recovery at 70 °C—significantly outperforming the base biosurfactant–polymer formulation without nanoparticles (80.94%) and brine flooding (58.34%) [116].
- Bio-based Alkali–Surfactant–Polymer (Bio-ASP) Formulations: Incorporating biosurfactants into the established ASP flooding framework creates an environmentally optimized Bio-ASP system. Adding a low-concentration of alkali (e.g., sodium carbonate) can generate in situ soaps with the biosurfactant to achieve ultra-low IFT. An IFT of 0.02 mN/m was achieved by Taher Al-Ghailani et al. [121], with 0.9% (*w*/*v*) alkali added into a 20-times diluted biosurfactant solution. A synergy effect was also observed in altering wettability and reducing the adsorption loss of the biosurfactant onto negatively charged rock surfaces. Consequently, Bio-ASP significantly improves chemical utilization efficiency, owing to these combined advantages, making it a highly promising EOR strategy [112,121].

### 4.3. The Synergistic Outcome and Potential

The rational design of synergistic formulations transforms biosurfactants from standalone wettability alteration agents into the core components of comprehensive EOR fluids. By concurrently addressing IFT reduction, wettability alteration, viscosity control, and in situ stability, such composites tackle the multifaceted barriers to oil displacement more effectively. Many core flooding tests have demonstrated that well-designed biosurfactant-based synergistic formulations can recover an additional 15% to 25% of OOIP over conventional waterflooding [122]. This approach not only amplifies the functional benefits of biosurfactants but also strengthens their economic and operational viability. Therefore, developing synergistic formulations is becoming a critical advancement in deploying microbial solutions for subsurface oil recovery.

## 5. Key Challenges for Biosurfactant Application in Subsurface Reservoirs

Despite their immense potential, the journey of biosurfactants from the controlled laboratory trails to successful field-scale application is fraught with following challenges.

### 5.1. Resilience Under Extreme Reservoir Environments

Biosurfactants in subsurface oil reservoirs are subjected to extreme conditions, such as high temperature, high salinity, variable pH. These hash aquatic environments pose distinct threat to the integrity and function of biosurfactants.

- High Temperature: In deep formations, reservoir temperatures can easily exceed 70 °C and even surpass 120 °C. Such heat can denature the proteinaceous components of lipopeptides or degrade glycolipid structures, leading to irreversible loss of surface activity. Therefore, it is necessary to screen and engineer thermotolerant strains. Encouragingly, many biosurfactants, especially lipopeptides from Bacillus species, display innate thermal robustness that often surpasses synthetic surfactants. Bo Wu et al. reported that lipopeptide produced by the Bacillus subtilis strain kept excellent surface activity at 120 °C and pH 5–12 [54]. Marcela Nunes Argentin et al. isolated bacterial strain Ar70C7-2 from a rock of a deep offshore Brazilian oil reservoir and obtained the produced biosurfactant, which showed splendid emulsification and IFT reduction ability and maintained its stability at temperatures ranging from −18 to 121 °C [3,123]. Surfactin from Bacillus tequilensis was also reported to show negligible decay in interfacial tension reduction after 10 days of aging at 90 °C [100].
- High Salinity: Formation water is often highly saline, with total dissolved solids frequently exceeding 100,000 ppm (10% salinity) and sometimes reaching saturation levels (>20%). High salt concentration can screen electrostatic repulsions, especially the presence of divalent cations (e.g., Ca^2+^, Mg^2+^), which can precipitate anionic biosurfactants by forming insoluble salts and removing them from the solution [37,43,114,124]. High ionic strength can also alter the HLB of the surfactant, thus impairing its performance in IFT reduction and wettability alteration. Luckily, like biosurfactants produced by thermophilic microorganisms can endure high temperature, biosurfactants produced by halophilic microorganisms often exhibit exceptional salt tolerance ability. Xiaotong Wang et al. obtained a biosurfactant from Bacillus halotolerans and demonstrated that it can maintain high activity in salinities up to 21% NaCl [125]. Notably, tolerance is not universal; the stability of common biosurfactants like rhamnolipid, for example, has been observed to decrease with increasing salinity [91]. Therefore, screening or engineering microbial strains specifically adapted to target reservoir conditions is crucial.
- pH Fluctuations: Reservoir pH can vary, though it is typically near-neutral to slightly alkaline. The activity of biosurfactants, particularly those with ionizable functional groups like carboxylates or amines, is pH-dependent. At low pH, for instance, the carboxylate groups of anionic biosurfactants become protonated, reducing their solubility in water and diminishing their surface activity [126,127]. The ideal biosurfactant should have a broad pH stability range that encompasses the conditions of the target reservoir.

### 5.2. Adsorption and Loss

A primary economic and technical challenge in biosurfactant flooding is the significant, non-productive loss of injected agents due to adsorption onto the vast mineral surface area of the reservoir rock. Once adsorbed, the surfactant is unavailable to reduce interfacial tension or alter wettability at the displacement front, drastically diminishing process efficiency and increasing cost. The extent of adsorption is governed by a complex interplay of rock–fluid–surfactant interactions, with several key factors determining the degree of loss.

(1) Rock Type and Mineralogy

The reservoir rock composition is the most critical factor, dictating the dominant adsorption mechanisms.

- Sandstone Reservoirs: Composed primarily of quartz, sandstone surfaces are negatively charged under typical reservoir conditions. This results in weak electrostatic adsorption of anionic biosurfactants (e.g., rhamnolipids) but strong attraction to cationic surfactants [75].
- Carbonate Reservoirs: Carbonates (e.g., limestone, dolomite) present a greater challenge. Due to the adsorption of crude oil components like asphaltenes, the rock surfaces are often oil-wet [128,129,130]. Therefore, wettability alteration is a key target for biosurfactants, but their high adsorption and loss remains a major problem. More critically, the surface charge of carbonates is highly pH-dependent. At typical formation brine pH, which is usually below the zero point of charge of calcite (8–9.5), carbonate surfaces are positively charged [131,132]. The strong electrostatic attraction between the positively charged rock surface and negatively charged biosurfactants leads to much higher adsorption losses.

(2) Biosurfactant Concentration

Adsorption is highly concentration dependent. Below the critical micelle concentration (CMC), adsorption onto rock surfaces increases with bulk concentration. Above the CMC, adsorption typically plateaus as micelles form in the solution, and as the chemical potential of free surfactant monomers stabilizes [133]. Operating near or above the CMC is therefore a key strategy to balance effectiveness and loss.

(3) Brine Chemistry

The ionic composition of the formation brine directly modulates electrostatic interactions. As earlier noted, high ionic strength can screen repulsive charges, potentially increasing adsorption. Nevertheless, divalent cations can bridge negatively charged surfactant head groups and mineral surfaces, exacerbating loss. The pH of brine poses significant influence on the rock surface and the biosurfactant, thus controlling the electrostatic driving force for adsorption.

Overcoming adsorption loss is essential for economic viability. Effective strategies focus on altering interfacial interactions as follows [134]:

- Use of Sacrificial Adsorbing Agents: Pre-injecting agents that competitively adsorb to mineral sites can reduce subsequent biosurfactant loss. Nanoparticles (e.g., SiO_2_, CaCO_3_, TiO_2_, ZrO_2_ and Al_2_O_3_) are especially attractive because of their high surface area and tunability [135,136,137,138]. For instance, the addition of colloidal SiO_2_ has been shown to reduce the adsorption of an anionic surfactant on sandstone by up to 61% [137].
- Formulation Engineering: Co-injecting polymers or alkali can alter system chemistry. Polymers can provide steric hindrance, while alkali increases pH and can reverse carbonate surface charge to negative, repelling anionic surfactants [114].
- Operational Optimization: Tailoring the ionic strength or pH of the injection brine to minimize electrostatic attraction between the biosurfactant and the target rock can significantly reduce adsorption.

### 5.3. Scalability and Economic Hurdles

The transition of biosurfactants from promising laboratory agents to a widely adopted technology for EOR is dependent on overcoming significant practical and economic hurdles. While the scientific mechanisms are well-established and field trials have demonstrated its technical feasibility in a variety of reservoir settings (see Table A1 in Appendix A.2), the economic viability of large-scale implementation remains the most critical barrier.

#### 5.3.1. The Challenge of High Production Costs

The most significant impediment is the high cost of biosurfactant production. They are currently estimated to be 2.5 to 10 times more expensive than commercial synthetic surfactants ($1–3/kg), with current production costs ranging from $5–20/kg [91,139,140,141]. This cost disparity stems from expensive refined substrates and energy-intensive downstream processing, which alone can account for 60–80% of total production costs [140,141].

To address this, research is heavily focused on practical, cost-reduction strategies. A primary approach is the use of cheap, renewable substrates, such as agro-industrial wastes (molasses, whey, plant oil residues), to drastically reduce production costs [142]. For instance, a techno-economic assessment by Sharma et al.’s team on surfactin production from soy hull demonstrated that process intensification strategies could lower production costs to $3.83/kg (a 42.2% reduction compared to the original process design), making it competitive with commercial sophorolipids ($2.95/kg) and rhamnolipids ($5.00/kg) [139]. This approach aligns with circular economy principles, offering both cost reduction and an improved environmental footprint [143].

Another key practical solution is the use of crude or partially purified biosurfactant preparations (e.g., cell-free supernatants or biocomplexes) for EOR [79,144,145]. Since the purity requirements for subsurface injection are far lower than for biomedical applications, eliminating costly purification steps can dramatically improve process economics.

Furthermore, strain improvement through genetic engineering (e.g., CRISPR-Cas9) and fermentation process optimization using statistical methods are crucial for maximizing yields from cheap substrates and creating hyper-producing strains [146,147].

#### 5.3.2. Economic Viability: Evidence from Field Applications

Despite higher chemical costs, the economic viability of biosurfactant-based MEOR is ultimately determined by the cost per incremental barrel of oil, or Unit Technical Cost (UTC). A comparative study was conducted on an alternative ASP formulation using a biopolymer report that, despite higher chemical costs, has a UTC ($16.80/bbl) that was comparable to a conventional system ($15.65/bbl) due to superior incremental oil recovery (22.4% vs. 18.8%) [148]. This demonstrates that superior performance can offset higher input costs.

This principle is validated by extensive field data, particularly from China, showing consistently high input–output ratios that underscore economic attractiveness:

- Shengli Oilfield: MEOR technology demonstrated significant long-term economic benefits. From 1998 to December 2019, field applications across 10 blocks achieved a cumulative incremental oil production of 30 × 10^4^ tons, with an average input–output ratio of 1:5.7 [149].
- Yanchang Oilfield: A field pilot was conducted in the Xingzichuan area, involving nine injection wells and 43 production wells, with a cumulative injection of 269.8 tons of the biosurfactant formulation. Following implementation, fluid production increased from 215 m^3^/d to 281 m^3^/d, with a well response rate of 90%, a 40% improvement in single-well productivity, and a cumulative incremental oil production of 1.36 × 10^4^ tons [143].
- Daqing Oilfield: Microbial huff ‘n’ puff treatments in 93 wells achieved a success rate of 74.2%, with an average incremental oil of 149 tons per well and a cumulative oil increase of 1.39 × 10^4^ tons. Importantly, the chemical cost per ton of incremental oil was only CNY 300 (approximately USD 43, based on an exchange rate of 7 CNY/USD), underscoring its cost-effectiveness [150].

The operational costs for MEOR are also highly competitive, estimated at $3–9 per barrel, compared to $10–53 for conventional chemical EOR [151]. Leveraging the ultra-low CMC and high dilution tolerance of biosurfactants, a huff ‘n’ puff treatment in 15 Bakken shale wells achieved an average incremental oil production of 1700 bbl per well, highlighting MEOR’s potential for enhancing oil recovery in unconventional reservoirs. Additional economic benefits include the ability of biosurfactants to reduce chemical surfactant usage by 30–50% in hybrid formulations, as demonstrated by a biochemical ASP system in Daqing which saved 25% on surfactant costs [143].

#### 5.3.3. Pathways to Large-Scale Implementation

The evidence strongly supports that biosurfactant-based MEOR can be successfully scaled. The path forward is not a single solution but a multi-pronged strategy: (1) continued cost reduction through waste utilization and crude product use [94]; (2) deployment first in harsh reservoirs where synthetics fail; and (3) integration into synergistic hybrid technologies (e.g., bio-ASP) to maximize performance and economic returns [152]. With ongoing innovation in biotechnology and process engineering, the practical and economic barriers to widespread implementation are surmountable, paving the way for a more sustainable approach to EOR.

## 6. Field Applications for Biosurfactant-Based Microbial-Enhanced Oil Recovery

The ultimate validation for any EOR technology lies in its field performance. Over the past several decades, MEOR, with biosurfactants as a core mechanism, has evolved from a laboratory concept to a technology deployed in numerous pilot and field-scale trials across the globe. The collective history and results from these field applications provide critical insights into the technology’s practical potential, operational challenges, and path forward.

### 6.1. Historical Overview and Implementation Strategies

Hundreds of MEOR field trials have been conducted worldwide, in countries including the USA, Canada, China, Romania, and Argentina [4]. Milad Safdel et al. conducted a comprehensive review of 47 field trials from 21 countries, and indicated that over 90% of these trials are successful, confirming the creditability of MEOR [153].

The application methods vary but typically fall into two categories:

- Huff-n-Puff (Cyclic Microbial Recovery): This is a single-well stimulation technique. A formulation of microbes and/or nutrients is injected into a production well. The well is then shut-in for a period (the “huff” or “soak” phase) to allow for microbial growth and biosurfactant production. Afterwards, the well is put back on production (the “puff” phase). The produced fluids contain mobilized oil. This method is relatively low-cost and is used to treat near-wellbore damage and stimulate individual wells. Some heavy oil wells that have undergone multiple rounds of steam stimulation are also attempting to apply microbial huff-and-puff technology to extend their economic life. Currently, the success rate of microbial huff-and-puff remains relatively low, at approximately 70% [80].
- Microbial Flooding: This is a reservoir-wide process analogous to a chemical flood, where a microbial/nutrient solution is injected continuously or in slugs into injector wells to displace oil towards producers. This approach is more complex but holds significant potential for enhancing ultimate recovery [38]. Field applications of the microbial huff-and-puff trial in China’s extra-low permeability reservoirs achieved an average incremental oil of 149 tons per well. A microbial flooding project in the Chaoyanggou Oilfield resulted in a cumulative incremental production of 60,000 tons, enhancing oil recovery by 4.95%. These successes, alongside systematic research on reservoir microbial ecology have demonstrated the viability of this method, despite its high cost of CNY 300 to 557 per ton of incremental oil [150].

### 6.2. The Core Operational Decision: Ex Situ vs. In Situ Microbial-Enhanced Oil Recovery

The success of biosurfactant-based MEOR field application depends on a critical operational decision: how to deliver the active agents to the target reservoir zone. There are two fundamental methods: ex situ and in situ strategies. Each method has its distinct formulation requirements, unique advantages, disadvantages, and profound implications for project design, cost, and risk management (see Table 3). The selection must be based on a comprehensive analysis of reservoir characteristics, inherent microbial ecology, and operational logistics.

Beyond the fundamental differences in implementation and formulation outlined above, in situ MEOR offers several profound advantages that extend well beyond simple oil mobilization. A significant, yet often overlooked benefit is its ability to improve the quality of the produced crude oil itself [4,45,127]. Direct microbial activity on the oil leads to beneficial compositional changes, including an increase in light alkanes (C < 20), a decrease in medium alkanes (C_20_–C_40_), biodegradation of high-MW hydrocarbons, cleavage of aromatic and phenolic rings, conversion of organic sulfur compounds, and enhanced emulsification. These processes collectively result in a lighter, less viscous, and higher-quality crude oil that is easier to produce and refine, adding substantial economic value to the process.

This qualitative improvement is coupled with the unparalleled “Low Cost” advantage of in situ MEOR. By utilizing inexpensive nutrients (e.g., molasses) and avoiding the high costs associated with fermentation, purification, and transportation of chemical or ex situ agents, its operating expenditure is significantly lower than that of chemical, thermal, or miscible EOR methods. Quantitative cost comparisons confirm this advantage: MEOR costs are estimated at $3–9 per barrel of incremental oil, whereas synthetic surfactant flooding ranges from $20–52/bbl, thermal EOR from $10–25/bbl, CO_2_ injection from $10–30/bbl, and polymer flooding from $10–20/bbl [37,166,167]. From an operational cost perspective, in situ MEOR is second only to conventional waterflooding, making it an economically attractive option for mature fields with marginal economics [37].

The realization of these benefits, however, hinges on a critical prerequisite: the careful selection of the right microbial candidate. Since the microbial community will utilize the resident crude oil as a primary carbon and energy source, the success of the process is inherently linked to the crude oil’s composition [14]. The natural variations in crude oil (e.g., paraffinic, naphthenic, asphaltic) mean that a single “super-strain” is unlikely to be universally effective. Consequently, a significant and essential part of developing an in situ MEOR project is the tailored selection of microbial strains or consortia. This requires extensive laboratory work to bioprospect for and match the metabolic capabilities of candidate microorganisms to the specific chemical fingerprint of the target reservoir’s crude oil, ensuring robust growth and the desired metabolic outcomes in situ [155].

### 6.3. Key Insights from Field Trials

Decades of field trials have yielded critical insights that are essential for designing successful future MEOR projects:

(1) Comprehensive reservoir assessment: A successful MEOR project requires a deep understanding of the reservoir’s geology, mineralogy, fluid properties, temperature, pressure, and indigenous microbial population, which is crucial for selecting the right MEOR strategy (in situ vs. ex situ) and the appropriate microbial strain or biosurfactant.
(2) Tailored Strain Selection: There is no one-size-fits-all microbe. The chosen strain (for in situ) or the production strain (for ex situ) must be robust enough to thrive or produce metabolites that are stable under the specific reservoir’s temperature, salinity, and pressure. Bioprospecting from environments analogous to oil reservoirs (e.g., hypersaline lakes, hydrothermal vents) is a promising source of suitable candidates [168].
(3) Targeted Nutrient Delivery: In in situ MEOR, the nutrient formulation directs the subsurface microbial metabolism. It must be designed to maximize the production of desired metabolites (like biosurfactants) while minimizing unwanted outcomes like excess biomass (which causes plugging) or hydrogen sulfide (H_2_S) from reservoir souring [169]. The inclusion of nitrate is a common strategy to outcompete sulfate-reducing bacteria [170].
(4) Robust Process Monitoring: An integrated monitoring program is key to timely and effective process optimization. This includes the monitoring of injection/production rates and pressures, chemical composition analysis of produced fluids (e.g., pH, biosurfactants, polymers, microbial counts), and fluid movement.

## 7. Overcoming Challenges and Future Perspectives

Scaling up biosurfactant-based MEOR requires solving current technical and economic challenges. Its future lies in the convergence with other EOR methods, driven by cost-effective production, synergistic hybrid technologies, and validated environmental benefits.

### 7.1. Cost-Effective Production: The Quest for Cheaper Feedstocks

The greatest challenge to the widespread application of ex situ biosurfactants is the production cost [171,172,173]. The solution lies in finding cheaper alternatives to purified substrates. Extensive research is focused on utilizing the following:

- Agro-Industrial Wastes: Substrates like molasses from sugar refining, crude glycerol from biodiesel production, whey from the dairy industry, and residues from vegetable oil processing are rich in carbohydrates and lipids, making them ideal feedstocks for many biosurfactant-producing microbes.
- Lignocellulosic Biomass: Developing microbial strains or consortia that can directly convert non-food plant biomass (e.g., corn stover, switchgrass) into biosurfactants would open up a vast and sustainable resource base.

This approach not only dramatically lowers production costs but also improves the overall environmental footprint of the technology.

### 7.2. Hybrid Technologies: The Power of Synergy

The future of EOR lies not in silver-bullet solutions but in intelligently combining different technologies to achieve synergistic effects.

- Biosurfactants and Low-Salinity Water (LSW) Flooding: LSW flooding is an emerging EOR technique where water with reduced salinity is injected into the reservoir. Low-salinity environment can induce wettability alteration in the rock surface towards a more water-wet state by modifying the ion-exchange equilibrium. This phenomenon is particularly pronounced in sandstone reservoirs containing clays. Combining LSW in MEOR can provide a favorable initial shift in wettability and a better environment for biosurfactant to function, thus lowering the consumption of biosurfactant and the overall cost [174,175].
- Biosurfactants and Smart Waterflooding: “Smart Water” is an extension of LSW. In smart waterflooding, the ionic composition (instead of only the total salinity) of the injected water is carefully customized to promote rock–fluid interactions. A smart water formulation could be designed to maximize the stability and activity of a co-injected biosurfactant, creating a highly optimized and targeted EOR fluid [176,177].

### 7.3. Environmental Impact and Lifecycle Assessment

A core advantage of MEOR is its perception as a “green” technology. Biosurfactants are biodegradable and have significantly lower toxicity than their synthetic counterparts, minimizing the risk of long-term contamination of groundwater aquifers. To move beyond qualitative claims, the field needs more rigorous and comprehensive Lifecycle Assessments (LCAs) [178,179,180,181]. These studies would quantitatively compare the full environmental footprint of biosurfactant-based MEOR against cEOR, considering everything from raw material extraction (or feedstock cultivation) and energy consumption during production to final disposal and long-term environmental fate. Such LCAs will be crucial for policy-making and for validating the environmental credentials of MEOR to regulators and the public [182].

### 7.4. Conclusions

Microbial biosurfactant is a bio-based eco-friendly solution to the global challenge of maximizing recovery from mature oil reservoirs. The primary mechanism of biosurfactant-based MEOR is the wettability alteration in the rock surface, particularly in aged, oil-wet formations. By adsorbing at the oil–rock interface and rendering the rock surface from oil-wet to water-wet, biosurfactants can lower the IFT, overcome the capillary forces, and mobilize the trap residual oil. Combining biosurfactants with biopolymers, alkali, nanoparticles, etc., and assisted with LSW flooding or smart waterflooding, MEOR is advancing from a laboratory idea to a prospective field application technology. This review has elucidated the mechanisms of this process, delineated the diverse and potent classes of biosurfactants, and explored the significant challenges posed by harsh reservoir conditions and economic constraints. To address these challenges, future research shall embrace innovations in bioprocessing, formulation science, and reservoir engineering. In this endeavor, by viewing the world’s diverse aquatic environments as a living library of biochemical solutions, this review can continue to develop MEOR not just as an effective method for enhanced oil recovery, but as a flagship example of how biotechnology can provide sustainable solutions to pressing industrial challenges, bridging the gap between microbial ecology and modern energy production.

## Figures and Tables

**Table 1:** Ultra-low interfacial tension values reported for key biosurfactants [56].

Biosurfactant	Interfacial Tension (mN/m)	Liquid Phase	Reference
Rhamnolipid PG201	4.5	Ekofisk crude oil	[57]
	0.08 at PH 5.4	dodecane benzene	[58]
Rhamnolipid Dyna 201	0.06 at PH 5.4 0.94 at PH 5.7	Isooctane benzene	[58]
Rhamnolipid	4	Crude oil	[59]
	1.32	hexadecane	[60]
Bacillus biosurfactant	4.5	hexadecane	[61]
Bacillus lipopeptide JF-2	0.006 at PH 6 0.023 at PH 7	Decane/5° NaC1	[62]
Mannosylerythritol lipid	0.1	Kerosene	[63]
Trehalose glycolipid	1.5	hexadecane	[64]
Lipopeptide Surfactin	0.2 2.03	Diesel oil	[65]
	2.03	dodecane	[66]

**Table 2:** Major classes of low-molecular-weight biosurfactants for MEOR applications.

Biosurfactant Class	Sub-Type Example	Producing Microorganism (Typical)	Key Structural Features	Properties and MEOR Relevance
Glycolipids	Rhamnolipids	Pseudomonas aeruginosa	One or two rhamnose sugar molecules linked to one or two β-hydroxy fatty acid chains. Anionic.	Excellent IFT reduction. Proven wettability alteration capability. High stability in a wide range of T, pH, and salinity [91].
	Sophorolipids	Starmerella bombicola (Yeast)	Sophorose (a disaccharide) linked to a long-chain hydroxy fatty acid. Can be lactonic (cyclic) or acidic (linear). Non-ionic/Anionic.	Good emulsifying properties. Production yields are often high. Potential for cost-effective EOR applications [92].
Lipopeptides	Surfactin	Bacillus subtilis	A cyclic lipopeptide of 7 amino acids linked to a C_12_-C_16_ β-hydroxy fatty acid chain. Anionic.	Extremely high surface activity; one of the most effective biosurfactants known. Reduces surface tension of water to ~27 mN/m [3]. Good thermal stability, but can be sensitive to divalent cations/high salinity [43].
	Lichenysin	Bacillus licheniformis	Similar structure to surfactin; cyclic heptapeptide linked to a β-hydroxy fatty acid. Anionic.	High thermal and pH stability. Particularly effective and stable in high-salinity environments, making it a strong candidate for offshore EOR [93].

**Table 3:** Comparison of in situ and ex situ MEOR strategies.

Feature	Ex Situ MEOR (Biosurfactant Injection)	In Situ MEOR (Microbial Injection)
Concept	Biosurfactants are produced in industrial fermenters, separated, and injected as a chemical solution into the reservoir [154].	A consortium of selected microorganisms and a nutrient package (e.g., molasses) are injected into the reservoir. Biosurfactants are produced downhole [155].
Formulation	Purified or semi-purified biosurfactant solution, often combined with biopolymers or other agents [156].	Microbial inoculum, carbon source (molasses, sugars), nitrogen/phosphorus sources, and minerals.
Advantages	High Control: Precise control over the type, concentration, and quality of the biosurfactant being injected [157,158]. Predictable: Process performance is easier to model and predict. No Bio-Plugging Risk: Avoids risks of uncontrolled biomass growth and reservoir souring [159].	Lower Cost: Avoid expensive downstream processing and purification. Deep Penetration: Microbes can travel deep into the reservoir and produce agents at the oil–rock interface where they are most needed [160]. Synergistic Mechanisms: Multiple EOR mechanisms (biosurfactants, biopolymers, acids, gases) function concurrently.
Disadvantages	High Cost: Fermentation and especially purification costs are a major economic barrier [91,115]. Adsorption Loss: Injected surfactant is subject to significant loss via adsorption onto rock surfaces [161]. Transport Issues: Ensuring the formulation reaches the target zone without degradation.	Uncontrollable: Hard to control microbial growth and metabolic activity in subsurface reservoir. Plugging Risk: Uncontrolled biomass growth can plug pore throats and damage permeability [162,163]. Reservoir Souring: Potential H_2_S production by sulfate-reducing bacteria [164]. Nutrient Transport: Poor nutrient distribution uniformity.
Best Suited For	High-temperature reservoirs where microbial survival is difficult; reservoirs where precise chemical control is desired; testing specific biosurfactant performance.	Reservoirs with low-moderate temperature (<80 °C); reservoirs with favorable geochemistry; cost-sensitive operations [165].

## Data Availability

No new data were created or analyzed in this study.

## Abbreviations

The following abbreviations are used in this manuscript:

ASP	Alkaline–surfactant–polymer
Bio-ASP	Bio-based Alkali–Surfactant–Polymer
cEOR	Chemical Enhanced Oil Recovery
CMC	Critical micelle concentration
EOR	Enhanced Oil Recovery
HLB	Hydrophilic–lipophilic balance
HTHS	High temperature, high salinity
LSW	Low-Salinity Water
MEOR	Microbial-Enhanced Oil Recovery
MW	Molecular weight
OOIP	Original oil in place
UTC	Unit Technical Cost

## Appendix A

### Appendix A.1. Methodology

This review was compiled through a comprehensive literature search conducted in major scientific databases, including Web of Science, Scopus, PubMed, (China National Knowledge Infrastructure), and Google Scholar (the latter used as a supplementary tool). The search employed keywords and combinations such as “microbial biosurfactants,” “MEOR,” “wettability alteration,” “biosurfactant EOR field trials,” “glycolipids oil recovery,” and “lipopeptides reservoir.” The search covered publications from 1980 to 2025, with particular emphasis on studies published in the last decade. We prioritized peer-reviewed original research articles, comprehensive review papers, and field trial reports. Articles were selected based on their relevance to biosurfactant mechanisms, reservoir applications, field performance data, and economic or scalability analyses. A total of 190 references were included in the final manuscript, representing the most significant contributions to the field”.

### Appendix A.2. Field Applications Summary

**Table A1:** Biosurfactant-based MEOR field applications, scale, and production performance.

Country	Oilfield/Block	Application Period	Application Process/Scale (and Biosurfactant Used)	Production Performance Results	Success/ Response Rate
USA	DuPont Test Block	2006–2012	Ex situ Microbial Flooding (Biosurfactant-based metabolites), 17 wells	Out of 17 wells, 15 responded; water cut reduced by 5%; oil production increased by 15–20% [183]	88.2%
	Bakken Shale	2021	Biosurfactant Huff ‘n’ Puff/15 wells	Average cumulative incremental oil of 1700 bbl per well; quick return on investment [184]	Successful
Oman	Eastern Reservoir	Recent	Bio-ASP Flooding/Core flood study (Lipopeptide biosurfactant–surfactin)	Achieved 32% additional oil recovery in Berea cores [121]	Lab Success
China	Daqing Chaoyanggou Oilfield	2004–2018	Ex situ Microbial Flooding (Surfactant-producing mixed culture)/9 injectors, 24 producers	Cumulative incremental oil: 60,000 t; recovery factor increased by 4.95%; water cut dropped from 46.8% to 40.3%; monthly oil production peaked at 843 t from 361 t [150]	70%
	Daqing Sanan Oilfield	2011	Indigenous Microbial Flooding (Surfactant-producing functional flora)/1 injector, 4 producers	Cumulative incremental oil: 6243 t; recovery factor increased by 3.93%; water cut dropped from 96.2% to 93.9% [150]	Successful
	Daqing Multiple Blocks	Recent	Biochemical ASP Flooding (Lipopeptides, Rhamnolipids Surfactant-producing microbes)/189 injectors, 203 producers	Saved 25% on surfactant costs; in the Sartu pilot (23 injectors, 33 producers), maximum water cut drop was 18%, recovery factor increased by 12.74% [143]	Successful
	Shengli Zhan 3 Block	2011–2019	Indigenous Microbial Flooding (Lipopeptides, Rhamnolipids)/3 injectors, 11 producers	Cumulative incremental oil: 61,300 t; recovery factor increased by 3.13% [80,185]	Successful
	Shengli Xin 68 Block	2016–2019	Indigenous Microbial Flooding/2 injectors, 6 producers	Cumulative incremental oil: 12,000 t; recovery factor increased by 2.98% [149]	Successful
	Shengli Binnan Shan 14 Block	2018–2020	Microbial + CO_2_ Huff ‘n’ Puff (Surfactant-producing microbes)/13 wells	Cumulative incremental oil: 12,000 t; Well SJSH14X60’s oil production increased from 0.5 t/d to 7 t/d [149]	90.5%
	Shengli SJSH10P2 Well	2019–2020	Microbial + Biosurfactant Huff ‘n’ Puff/Single well	Peak oil production: 8.6 t/d; oil–gas ratio reached 1.56 (compared to 0.55 for the last steam stimulation cycle) [149]	Successful
	Shengli Luo 9 Shi 1 Block	2020–2021	Microbial Polysaccharide + Fermentation Broth Huff ‘n’ Puff (Rhamnolipids)/4 wells	Cumulative incremental oil: 2730 t [146]	4 out of 4 wells responded
	Yanchang Xingzichuan Oilfield	Recent	Biosurfactant-based Compound Flooding/9 injectors, 43 producers	Cumulative incremental oil: 13,600 t; well response rate: 90%; single-well productivity increased by 40% [143]	90%
